# Obsessive–compulsive disorder: An interface with possible psychotic features

**DOI:** 10.4103/0019-5545.55946

**Published:** 2005

**Authors:** K.Nagaraja Rao, C.Y. Sudarshan, Preethi Pai

**Affiliations:** * Professor and Head, Department of Psychiatry, J.J.M. Medical College, Davangere; ** Reader, Department of Psychiatry, J.J.M. Medical College, Davangere; *** Postgraduate Student, Department of Psychiatry, J.J.M. Medical College, Davangere

(The following case discussion is a part of a grand round conducted in the Department of Psychiatry, JJM Medical College, Davangere, and reflects a group process with discussion among the PG students and consultants. The abbreviations used are C1 and C2 for Consultants; PP for the presenting PG student; PG1, PG2 for the participating PG students.)

## A. CASE PRESENTATION

### History

Mr A, an unmarried Hindu male aged 20 years, and a resident of Davangere, was unemployed, had studied up to standard XI and came from a low socioeconomic background. He came to the hospital with his mother and brother with the complaints of fear of going out of the house, repetitive uncontrolled thoughts and repetitive acts of two-and-a-half years' duration.

#### History of present illness

The patient was apparently asymptomatic till two-and-a-half years back. One day, he was called to be a witness in a criminal case that had occurred near his residence, despite his ignorance of the case. He was threatened with trouble if he declined to be a witness. The patient neither gave any evidence nor faced any problem. However, since then, the patient has been scared to go out of the house alone. He fears that some harm may befall him. He cannot specify the nature and source of the harm. The patient became so fearful of going out of the house that, unless accompanied, he would pass urine in the house.

The patient's relatives and parents had suggested that someone had performed black magic on the patient and, therefore, he had got into trouble. The patient started believing this and continues to believe it. He gets repetitive thoughts about black magic being done on him. He gets thoughts that to ward off the ill-effects of black magic he should join the people who perform it, pay them Rs 10,000–15,000 and perform acts such as running around the stadium 50 times. He realizes that it is not possible for him to act on these thoughts. Though a very religious person, he has been unable to pray since the past 2 years as, whenever he prays ‘do good to me’, these words are replaced by ‘do bad to me’. This distresses him a lot. He knows that these thoughts are his own and, though they are distressing, he cannot stop them. Because of these thoughts he cannot concentrate on watching TV, reading, etc. Because of repetitive thoughts, the patient feels very irritated, distressed and sad, and feels like ending his life. There are no symptoms of sadness and suicidal ideas in the absence of repetitive thoughts.

He performs certain rituals to prevent the effects of the alleged black magic. These include buttoning his shirt, wearing trousers, touching objects and crossing the threshold nine times each, washing his hands three times and dividing the last morsel of food into four parts before eating it.

The patient has been sleeping well and his appetite is normal. He has no history of crying spells, early morning awakening, seeing or hearing things that do not exist, violent behaviour, substance use or abuse.

Initially, the patient was taken to a temple for 4–5 months, but the symptoms did not subside. He was then shown to a psychiatrist on an outpatient basis. He took treatment for one month. As the symptoms did not subside he discontinued treatment and whenever the symptoms increased, he would stay in a temple and work there, which made him feel better temporarily. Since the past one month, the symptoms had increased in intensity and hence he was brought to the hospital.

#### Past history

There was no significant medical or psychiatric past history.

#### Family history

The patient stays in a joint family and is the first among three siblings. There is no family history of overt psychiatric illness, major medical illness, substance abuse, suicide or epilepsy.

#### Personal history

The patient discontinued studies in standard XI as his symptoms interfered with his studies. Presently, he is unemployed.

#### Pre-morbid personality

The patient had few friends and preferred his own company. Communication with family and friends was limited, and he was not employed. He would sit by himself and watch TV. His general mood was restricted and he had no rapid mood swings. His moral, social, religious and health standards were high. General and systemic examination was unremarkable.

### Mental status examination

#### 1. General appearance and behaviour

The patient is well groomed and dressed appropriately for his socioeconomic status. Psychomotor activity is normal. He makes eye contact, and rapport is easily established. He is cooperative and communicative, and has no tics or mannerisms.

#### 2. Speech

The patient's speech is spontaneous, with normal reaction time and pitch. The tone varies according to the situation. Speech is relevant and coherent, the answers appropriate and understandable.

#### 3. Mood

Subjectively, the patient expresses anxiety. Objectively, he appears anxious. Reactivity is present. His mood is appropriate for the situation and is congruent with thought. There is no lability. The range is restricted.

#### 4. Thought

*Stream*—Normal, goal-directed, no thought block, circum-stantiality, tangentiality, perseveration, flight of ideas or stereotypy. *Form*—Well-structured, understandable, no loosening of association, poverty of production or content of speech seen.*Possession*—Recognizes his thoughts as his own. Patient gets obsessions about black magic and compulsions about performing some acts. *Content*—Ideas of persecution are present.

#### 5. Perception

The patient has no hallucination, illusion, derealization, depersonalization, hyper- or hypoaesthesia.

#### 6. Attention

The patient could perform the digit forward 5, and digit backward 5 tests without error and prompting. The conclusion is that his attention is easily aroused and concentration adequate.

#### 7. Orientation

*Time*—The patient could tell the time of the day, date, month of the year. *Place*—He could tell where he was and the name of the town. *Person*—He gave his name, age, occupation and other details correctly; he correctly identified the doctor, nurse and his brother. The conclusion is that the patient is well-oriented to time, place and person.

#### 8. Memory

*Immediate*—The patient had no difficulty in doing the 5 digit forward and 5 digit backward tests. *Recent*—He could correctly remember what he ate for breakfast and when he came to the hospital. *Remote*—He remembered the time of his sister's marriage correctly. The conclusion is that the patient's memory is intact.

#### 9. Intelligence

*General fund of knowledge:* The patient could tell the name of the Chief Minister of the state, Prime Minister of the country, and knew about a major sports event that was going on. *Mathematical ability:* Simple and carryover calculations were easily performed. *Abstraction:* He knew the similarities and differences between a chair and a table, a potato and a stone. Abstract meanings of proverbs were correctly explained. The conclusion is that his intelligence is adequate for his socio-educational status.

#### 10. Judgement

*Test:* On being asked a question as to what he would do if his house caught fire, he said that he would try to put it out. *Social:* He was well behaved in the ward and followed ward routine properly. *Personal:* On being asked about his future plans, the patient said that he would like to finish his education and get a job. The conclusion is that his judgement is intact.

#### 11. Insight

The patient recognizes that he has an illness but he ascribes it to external factors, i.e. black magic. His level of insight is 3.

### Diagnosis

Axis I: Obsessive–compulsive disorder

Axis II: Not yet diagnosed, planning to evaluate

Axis III: Nil

Axis IV: Nil

Axis V: 51%–60%

## B. CLINICAL DETAILS

**PG1:** What are obsessions?

**PP:** Obsessions are persistent, recurrent, intrusive, inappropriate thoughts, ideas, impulses or images which cause anxiety or distress and are recognized as one's own thoughts and are resisted. These are unrelated to problems of situations.

**C2:** What is this patient's orderly behaviour called?

**PP:** It is called obsessional rituals or compulsive ceremonial. These are senseless behaviours repeated in a systematic way according to a prearranged plan or routine to avoid the danger associated with spontaneity. This is a form of systematization. These include counting in a special way or repeating words for a certain time, washing the hands many times, laying out clothes in a complicated way before dressing. If the desired sequence and numbers cannot be achieved, the whole process is repeated again. Sometimes others are pressurized to follow the patient's system in an effort to ensure their validity.

**C1:** You said that when he prays ‘do good to me’ the thought ‘do bad to me’ comes to the patient's mind. What is this called?

**PP:** This is called contradictory thinking, in which the thoughts that come to the patient are the opposite of what he intends to think.

**C2:** What happens if the patient controls his thoughts or stops his actions?

**PP:** If the patient tries to control his thoughts or actions, he feels scared that he may be harmed due to black magic.

**C1:** Is it magical thinking? Define magical thinking.

**PP:** Yes. He believes that if he stops thinking and performing some specific actions, harm would ensue and his thoughts and actions prevent the occurrence of such harm. Magical thinking is a form of dereistic thinking in which thoughts, words or actions assume the power to cause or prevent events. The individual feels that an event occurs or is prevented in the external world by merely thinking about it, without intermediate physical actions.

**PG2:** Why not consider delusion?

**PP:** The patient knows that his thoughts are irrational and intrusive but cannot control them. He also wants his thoughts and actions to stop. Whenever there is a respite from his obsessions and compulsions he has no functional abnormality. His routine functioning is affected by his obsessions and compulsions. Hence, delusion is not considered.

**C1:** I would like all of you to note at this stage that a phenomenological distinction between obsessions and delusions/overvalued ideas is usually easy. However, in some cases the picture is confusing. This patient feels that black magic has been performed on him. At first sight, it appears to be a delusion/overvalued idea. However, further exploration reveals the true nature of the phenomenon, i.e. an obsession. The patient recognizes the thought as his own, finds it distressing and irrational, and tries to resist it. This can be conceptualized as a religious obsession and is best seen as an atypical presentation.

## C. PSYCHOPATHOLOGY AND DIAGNOSIS

**PG1:** Can OCD progress to psychosis?

**PP:** Yes, in some cases, the patient stops resisting obsessional thoughts and starts believing in them. This is obsessional psychosis, which was described by Insel and Akiskal in 1986. In this condition, there is impaired reality and one believes that the thoughts are real, obsessions are reasonable and compulsive behaviour is necessary.

**C2:** Let me point out at this stage that we must be aware of the interface between OCD and psychosis, which is a source of diagnostic dilemmas. Obsessions as we all know are characteristically seen in OCD. They are not uncommon in patients with psychosis where the two can coexist (i.e. the patient has insight into the obsessions) or they can be seen as the manifestations of an underlying delusion, where insight is absent. In the entity OCD with poor insight, which can be seen in patients with chronic OCD, the senselessness and resistance of the obsessions is gone. However, this is not the same as OCD and psychosis. In OCD with poor insight the patient harbours no delusions. It is important for us to distinguish between these phenomena as the diagnosis and consequently treatment will vary. Finally, it is worth remembering that obsessive–compulsive behaviour can also be seen in the prodrome of psychotic states as a transient phenomenon.

**PG2:** What about ‘obsessional psychosis’ in current classificatory systems?

**PP:** DSM-IV uses an OCD specifier ‘with poor insight’, wherein obsessions and compulsions are not recognized as excessive and unreasonable. Further, in DSM-IV, it is recognized that the beliefs that underlie the obsessions in OCD can be delusional and, in such cases, it is suggested that an additional diagnosis of delusional disorder or psychosis not otherwise specified (NOS) may be used.

**C2:** How will you distinguish between OCD and psychosis with symptoms of obsession and compulsion?

**PP:** In OCD, the patient acknowledges the unreasonable nature of his symptoms. These are ego-dystonic to him. The repeated thoughts are intrusive, unwanted and resisted. Symptoms centre around obsessions and compulsions such as washing hands repeatedly, checking locks, etc. Reality testing is maintained. In psychosis, the patient does not recognize his symptoms as unreasonable. They are ego-syntonic to him. Delusional ideas are firmly held and are considered true and not resisted. Symptoms of an accompanying psychotic disorder such as hallucinations, abnormal behaviour, etc. are present. Reality testing is altered.

**C1:** What rating scales are used to rate OCD?

**PP:** The Maudsley Obsessional Compulsive Interview (MOCI), Yale Brown Obsessive Compulsive Scale (Y–BOCS), Leyton Obsessional Interview, Padua Obsessional Interview, Hopkins Symptom Check List are the scales commonly used to rate OCD.

**C2:** Is there any scale to measure insight in OCD?

**PP:** Insight in OCD can be measured by the seven-item Brown Assessment Beliefs Scale (BABS) developed by Eisen *et al.*

## D. MANAGEMENT OPTIONS

**C1:** How do you treat OCD biologically?

**PP:** Biological treatment is mainly pharmacotherapy. Clomipramine, fluvoxamine, fluoxetine, paroxetine and sertraline are commonly used drugs. Other drugs are imipramine, citalopram, moclobemide. In OCD, antidepressants are used in higher doses than in depressive disorders. In some cases, lithium, venlafexine, phenelzine, L-tryptophan and clonazepam have been used.

**C2:** What is the minimum trial period of a drug for the treatment of OCD? How do you treat non-responders?

**PP:** A clinical trial of 10 weeks is the minimum. Of the patients receiving pharmacotherapy, 40%–60% are respon-ders. Non-responders to one drug may respond to another. In some cases, augmentation strategies and ECT can be used. Antipsychotic drugs in low doses may be added when there is a co-morbid schizotypal personality disorder.

**PG2:** What are the augmentation strategies?

**C2:** Augmentation drugs used in OCD are (1) D-fenfluramine with clomipramine; (2) the 5-HT_A1_ agonist buspirone; (3) lithium, which is known to increase presynaptic 5-HT release in the brain; (4) clonazepam, a benzodiazepine with 5-HT properties; (5) trazodone, a 5-HT-2 and alpha-adrenergic blocker; (6) antipsychotics such as haloperidol and pimozide with an SSRI, especially when there is a co-morbid schizotypal personality disorder; (7) augmentation of SSRIs with gabapentin and phosphotidyl inositol are under study.

**C1:** As we have discussed, serotonin reuptake inhibitors are the mainstay in the treatment of OCD in conjunction with non-pharmacological measures. The role of antipsychotics in OCD is worth mentioning. They are useful when an independent psychotic disorder has been established along with OCD. They are not used in patients of OCD with poor insight. They are also useful as augmenting agents in partial responders to serotonin reuptake inhibitors.

**PG2:** What are the psychological treatments available?

**PP:** Psychological treatment includes behavioural therapy (BT), cognitive–behavioural therapy (CBT) and other psychotherapies. (i) BT is given in the form of exposure techniques, response prevention, blocking and punishing techniques. (ii) In CBT, initially irrational beliefs are distanced by diversion; later critical evaluation and rational criticism are taught. (iii) Other types of psychotherapy that are useful are supportive, insight-oriented psychotherapy, short-term psychotherapy, classical psychoanalysis and short-term dynamic psychotherapy.

**C2:** What is the prognosis in cases of OCD?

**PP:** OCD is usually chronic with a severity that waxes and wanes. About 20%–30% have marked improvement, 40%–50% have moderate improvement and 20%–40% remain the same or get worse. About 90% will require maintenance treatment. About 10% have a malignant, deteriorating course. About one third of patients will have a major depressive disorder. Suicide is a risk for all patients.

**C1:** What are the poor prognostic factors in OCD?

**PP:** These are onset in childhood, yielding compulsions, bizarre compulsions, need for hospitalization, coexisting major depressive disorder, delusional beliefs, presence of overvalued ideas, and presence of a personality disorder.

## E. SUMMARY

The case described and discussed above highlights a few clinically important issues. Although the patient was diagnosed as a case of OCD, the presentation is somewhat unusual. Fear of harm and thoughts about black magic usually suggest that psychotic states should also be considered and ruled out. The detailed discussion brings out the form of the symptom which helps make a diagnosis of OCD. There are some important issues which need to be considered in this and every such patient of OCD. First and foremost, a phenomenological analysis of the symptoms is important. This will lead to a correct diagnosis of the case. Second, one needs to remember the interface that exists between OCD and the group of psychotic disorders. This is often a source of confusion and, on occasion, leads to incorrect management. Finally, it is important to remember that antipsychotics have a limited role in OCD and are best limited to the situations mentioned in the discussion.
